# Control of Microbial Sulfide Production with Biocides and Nitrate in Oil Reservoir Simulating Bioreactors

**DOI:** 10.3389/fmicb.2015.01387

**Published:** 2015-12-08

**Authors:** Yuan Xue, Gerrit Voordouw

**Affiliations:** Petroleum Microbiology Research Group, Department of Biological Sciences, University of CalgaryCalgary, AB, Canada

**Keywords:** sulfate-reducing bacteria, nitrate, biocide, synergy, bioreactor

## Abstract

Oil reservoir souring by the microbial reduction of sulfate to sulfide is unwanted, because it enhances corrosion of metal infrastructure used for oil production and processing. Reservoir souring can be prevented or remediated by the injection of nitrate or biocides, although injection of biocides into reservoirs is not commonly done. Whether combined application of these agents may give synergistic reservoir souring control is unknown. In order to address this we have used up-flow sand-packed bioreactors injected with 2 mM sulfate and volatile fatty acids (VFA, 3 mM each of acetate, propionate and butyrate) at a flow rate of 3 or 6 pore volumes (PV) per day. Pulsed injection of the biocides glutaraldehyde (Glut), benzalkonium chloride (BAC) and cocodiamine was used to control souring. Souring control was determined as the recovery time (RT) needed to re-establish an aqueous sulfide concentration of 0.8–1 mM (of the 1.7–2 mM before the pulse). Pulses were either for a long time (120 h) at low concentration (long-low) or for a short time (1 h) at high concentration (short-high). The short-high strategy gave better souring control with Glut, whereas the long-low strategy was better with cocodiamine. Continuous injection of 2 mM nitrate alone was not effective, because 3 mM VFA can fully reduce both 2 mM nitrate to nitrite and N_2_ and, subsequently, 2 mM sulfate to sulfide. No synergy was observed for short-high pulsed biocides and continuously injected nitrate. However, use of continuous nitrate and long-low pulsed biocide gave synergistic souring control with BAC and Glut, as indicated by increased RTs in the presence, as compared to the absence of nitrate. Increased production of nitrite, which increases the effectiveness of souring control by biocides, is the most likely cause for this synergy.

## Introduction

Souring of produced oil, water, and gas is caused by production of sulfide by sulfate-reducing bacteria (SRB). Sulfide is present as H_2_S in all three phases and also as HS^−^ and as S^2−^ in the aqueous phase, depending on pH (Khatib and Salanitro, 1997; Gieg et al., 2011). Souring must be controlled due to the negative effects of hydrogen sulfide (H_2_S) on oil and gas quality (Vance and Thrasher, 2005) and due to increased risks of sulfide on health and safety (Beauchamp et al., 1984) and on biocorrosion of carbon steel infrastructure (Enning and Garrelfs, 2014). Souring can be controlled by the application of biocides or of nitrate (Davidova et al., 2001; Bødtker et al., 2008) with biocides being used mostly for control in above-ground infrastructure (e.g., tanks and pipelines) and nitrate being used mostly for control of souring in the reservoir. Other forms of control, like the use of bacteriophages to eliminate specific SRB, have also been advocated (Summer et al., 2011).

Biocides are organic chemicals designed to kill a broad spectrum of microorganisms. This broad spectrum activity, the possible persistence of biocides in the environment and the economics of biocide use all necessitate the choice of an optimal strategy that minimizes biocide use for a given application (Bradley et al., 2011; McGinley et al., 2011). The chemical structures and modes of action of some biocides, used in the oil industry and in this study, are indicated in Table 1. Glutaraldehyde (Glut) and tetrakishydroxymethyl phosphonium sulfate (THPS) are chemically-reactive biocides that kill microbes by irreversible chemical reactions, which inactivate the biocide. In contrast, benzalkonium chloride (BAC) and cocodiamine are physically-reactive biocides that kill bacteria by membrane disruption and cell lysis. These biocides remain active and toxic. THPS has no long alkyl chain R, like BAC and cocodiamine, so it is not also a physically-reactive biocide.

**Table 1:** **Some biocides commonly used in the oil industry, which were used in this study**.

**Biocide**	**Chemical structure**	**Mode of action**	**References**
Glutaraldehyde (Glut)	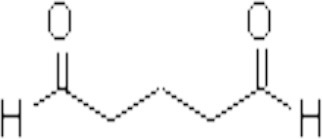	Chemically-reactive: aldehyde groups cross-link amino-groups in proteins and nucleic acids	Gorman et al., 1980; Bartlett and Kramer, 2011; McGinley et al., 2011
Benzalkonium chloride (BAC)	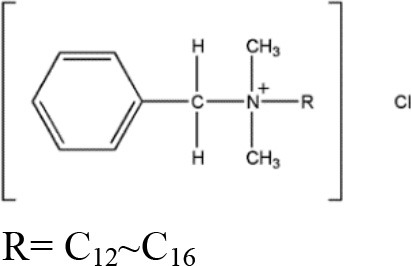	Physically-reactive: quaternary ammonium cationic surfactant, the long alkyl chain R solubilizes cytoplasmic membranes and causes cell lysis	Ferrer and Furlong, 2001; Ioannou et al., 2007; Ferreira et al., 2011; Oh et al., 2014
Cocodiamine	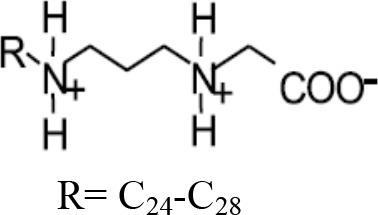	Same as BAC	Greene et al., 2006
Tetrakis hydroxymethyl phosphonium sulfate (THPS)	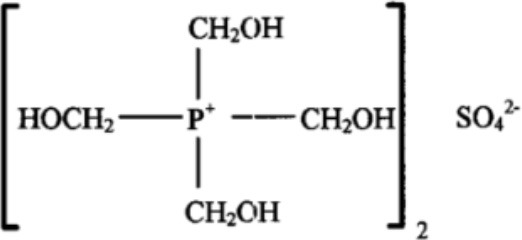	Reacts to denature proteins; damages membranes, interrupting proton flux and the ADP-ATP energy cycle; inhibits sulfate reduction by SRB	Jones et al., 2010, 2012

Souring control by nitrate injection has been extensively studied in the laboratory (Myhr et al., 2002; Hubert et al., 2003; Coombe et al., 2004; Grigoryan et al., 2008; Callbeck et al., 2011) and in the field (Jenneman et al., 1999; Sunde et al., 2004; Bødtker et al., 2008; Voordouw et al., 2009). Its mechanism involves the biocompetitive exclusion of SRB by heterotrophic nitrate-reducing bacteria (hNRB) (Davidova et al., 2001; Thorstenson et al., 2002; Hubert and Voordouw, 2007), the inhibition of SRB by nitrite (Reinsel et al., 1996; Sturman et al., 1999; Myhr et al., 2002; O'Reilly and Colleran, 2005) and the direct oxidation of sulfide with nitrate by sulfide-oxidizing NRB (soNRB) (Nemati et al., 2001a; Voordouw et al., 2002; Greene et al., 2003; Hubert et al., 2003). Compared with biocides, nitrate is cheaper, not broadly and persistently toxic, highly soluble in water and compatible with other chemicals. In spite of these advantages and its successful application in high temperature oil reservoirs, souring control in low-temperature oil reservoirs by continuous nitrate injection is more difficult (Voordouw et al., 2009). Following initial decreases, the concentration of produced sulfide recovered to pre-nitrate injection levels. This recovery was proposed to be due to the formation of zones of NRB in the near injection wellbore region (NIWR) and of SRB deeper in the reservoir (Voordouw et al., 2009; Callbeck et al., 2011). With excess electron donors, as expected in an oil field, and a favorable temperature in the deeper zone, SRB will continue to produce sulfide to cause souring with continuous supply of sulfate from injection water. Thus, souring control with nitrate can be transient in low-temperature reservoirs.

This paper addresses the question whether souring control by continuous injection of nitrate under low temperature conditions can be improved by combining this with pulsed injection of biocides. The use of biocides to prevent souring in reservoir-simulating bioreactors has not been extensively explored. We start this study, therefore, with a determination of the effectiveness of biocides in preventing souring in bioreactors in the absence of nitrate as a prerequisite for the evaluation whether continuous injection of nitrate and pulsed injection of biocides can be synergistic.

## Materials and methods

### Media and enrichment cultures

CSBA medium with volatile fatty acids (VFA, 3 mM each of acetate, propionate and butyrate) and either 2 mM sulfate (CSBA-S) or 2 mM sulfate and 2 mM nitrate (CSBA-SN) were used (Hubert et al., 2003; Callbeck et al., 2011). Sulfate and nitrate were added as the sodium salts. VFA are widely present in oil field produced waters and are easily oxidized by oil field hNRB and SRB. SRB in enrichments from the Medicine Hat Glauconitic C (MHGC) field (Voordouw et al., 2009) do not use acetate and incompletely oxidize propionate to acetate and CO_2_ and butyrate to two acetate. Under these conditions 12 mM acetate can be formed from the incomplete oxidation of 3 mM VFA (Grigoryan et al., 2008). hNRB use all VFA components with similar kinetics to reduce nitrate to N_2_ with nitrite as an intermediate (Grigoryan et al., 2008; Callbeck et al., 2011). The equations shown in Table 2 indicate that 1.33 mM propionate and 2 mM butyrate can each reduce 1 mM sulfate (equations 1 and 2), whereas 0.42 mM acetate, 0.24 mM propionate, and 0.17 mM butyrate suffice to each reduce 0.67 mM of nitrate. Hence, 3 mM VFA has sufficient reducing power to completely reduce 2 mM nitrate and 2 mM sulfate, which would require 0.42 mM acetate, 1.57 mM propionate and 2.17 mM butyrate, respectively, when equations 1–5 apply. If hNRB also reduce nitrate initially through incomplete oxidation of propionate and butyrate, then 0.83 mM propionate and 1.25 mM butyrate would be used to reduce 1 mM nitrate each (equation 6 and 7, respectively), leaving 2.17 mM propionate and 1.75 mM butyrate for sulfate reduction, which would still be enough for the complete reduction of both nitrate and sulfate. Hence, 3 mM VFA represents an excess of electron donors in both CSBA-S and in CSBA-SN medium. The headspace of all media was gassed with 90% (vol/vol) of N_2_ and 10% CO_2_ (N_2_-CO_2_), as described by Callbeck et al. (2011). Initial SRB enrichments used 10 mL of MHGC produced water and 90 mL of modified CSBA-S medium, containing 10 mM sulfate and 8 mM VFA in a 150 mL serum bottle with an N_2_-CO_2_ headspace. The culture was inoculated into bioreactor columns after two transfers in modified CSBA-S medium (Callbeck et al., 2011).

**Table 2:** **Stoichiometries for microbially-mediated oxidation of VFA by sulfate or nitrate^a^**.

${\text{SO}}_{4}^{2-}$	Propionate	${4\text{C}}_{3}{\text{H}}_{5}{\text{O}}_{2}^{-}{\;+\;3\text{SO}}_{4}^{2-}{\;+\;3\text{H}}^{+}\;\to {\text{ 4C}}_{2}{\text{H}}_{3}{\text{O}}_{2}^{-}{\;+\;3\text{HS}}^{-}{\;+\;4\text{CO}}_{2}{\;+\;4\text{H}}_{2}\text{O}$	(1)
	Butyrate	$2{\text{C}}_{4}{\text{H}}_{7}{\text{O}}_{2}^{-}{\;+\text{ SO}}_{4}^{2-}\;\to 4{\text{C}}_{2}{\text{H}}_{3}{\text{O}}_{2}^{-}{\;+\text{ HS}}^{-}{\;+\text{H}}^{+}$	(2)
${\text{NO}}_{3}^{-}$	Acetate	${5\text{C}}_{2}{\text{H}}_{3}{\text{O}}_{2}^{-}{+8\text{NO}}_{3}^{-}{+13\text{H}}^{+}\to {10\text{CO}}_{2}{+\text{4N}}_{2}{+15\text{H}}_{2}\text{O}$	(3)
	Propionate	${5\text{C}}_{3}{\text{H}}_{5}{\text{O}}_{2}^{-}{+14\text{NO}}_{3}^{-}{+19\text{H}}^{+}\to {15\text{CO}}_{2}{+7\text{N}}_{2}{+22\text{H}}_{2}\text{O }$	(4)
	Butyrate	${5\text{C}}_{4}{\text{H}}_{7}{\text{O}}_{2}^{-}{+20\text{NO}}_{3}^{-}{+25\text{H}}^{+}\to {20\text{CO}}_{2}{+10\text{N}}_{2}{+30\text{H}}_{2}\text{O}$	(5)
	Propionate	${5\text{C}}_{3}{\text{H}}_{5}{\text{O}}_{2}^{-}{+6\text{NO}}_{3}^{-}{+6\text{H}}^{+}\to {5\text{C}}_{2}{\text{H}}_{3}{\text{O}}_{2}^{-}{+3\text{N}}_{2}{+5\text{CO}}_{2}{+8\text{H}}_{2}\text{O}$	(6)
	Butyrate	${5\text{C}}_{4}{\text{H}}_{7}{\text{O}}_{2}^{-}{+4\text{NO}}_{3}^{-}\to {10\text{C}}_{2}{\text{H}}_{3}{\text{O}}_{2}^{-}{+2\text{N}}_{2}{+2\text{H}}_{2}{\text{O}+\text{H}}^{+}$	(7)

a *Calculated values for ΔG^0^′ (kJ per mol of sulfate or nitrate reduced) based on data by Thauer et al. (1977) were: (1) -50, (2) -56, (3) -495, (4) -496, (5) -496, (6) -497, and (7) -500*.

### Bioreactor setup and establishment of SRB biofilms

**Figure 1:**
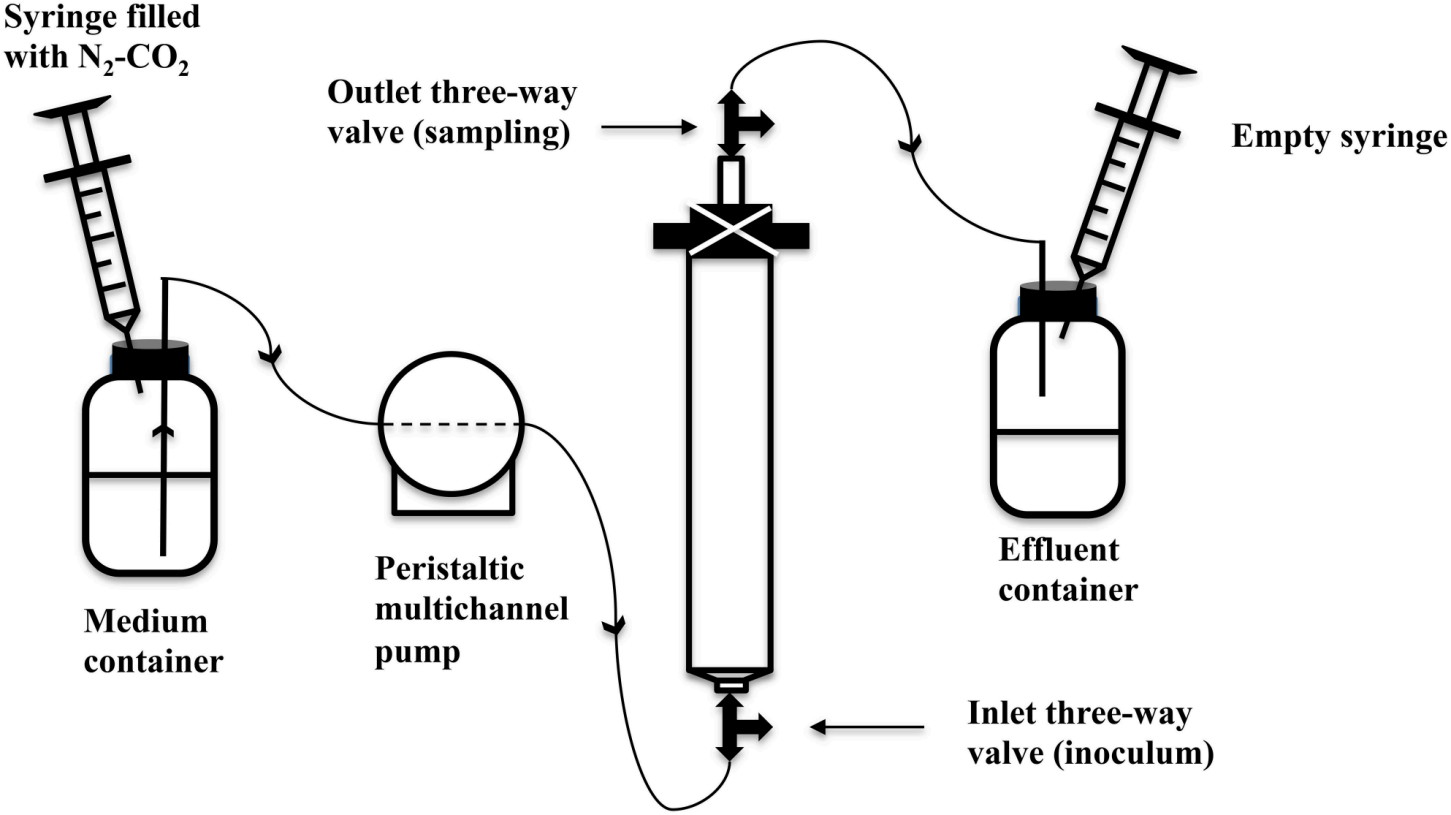
**Schematic of up-flow sand-packed bioreactor system modeling a souring oil field subjected to injection with nitrate and pulses of biocide**. Note that the actual flow direction in the field may be horizontal between the injection well and the production well. In an upflow bioreactor the inlet is equivalent to the injector and the outlet is equivalent to the producer.

Either plastic (60 mL, 12.2 × 2.7 cm) or glass (30 mL, 9.8 × 2 cm) syringes without piston were used as bioreactor columns. These were packed from the bottom to the top with a 1 mm layer of glass wool, a 3 mm polymeric mesh, sand (Sigma-Aldrich, 50–70 mesh particle size), a 3 mm polymeric mesh and a 1 mm layer of glass wool (Callbeck et al., 2011; Xue et al., 2015). The packed columns were closed with a rubber stopper perforated with a syringe needle, using zip ties on the outside. Three-way Luer-Lock valves were connected to the bottom inlet and the top syringe needle outlet to allow sampling of the influent and effluent streams. The packed and assembled dry columns were autoclaved. A peristaltic multichannel pump (Minipuls-3, 8-channel head, Gilson Inc.) was used to deliver water into the columns. PVC extension tubing (ID = 0.76 mm, Gilson, F117956) was used with PVC calibrated tubing (ID = 0.76 mm, Gilson, F117936) being used in the pump. SRB enrichment was inoculated from the bottom three-way Luer-Lock valve, while samples were taken from the effluent valve. The pore volume (PV) of the packed columns was determined by the weight difference between the column saturated with sterilized water and the dry column. Porosity was calculated as the fraction of PV over the total volume of the column. Anaerobic, sterile CSBA-S medium was then pumped from the medium container into the columns with effluent being collected in stoppered serum bottle effluent containers. The medium containers were fitted with 60 mL plastic syringes with piston, which were filled with N_2_-CO_2_, whereas the effluent containers were fitted with initially empty 60 mL plastic syringes with piston. This allowed continuous balancing of pressure (Figure 1). SRB enrichment (0.5 PV) was then inoculated into the bioreactor columns through the bottom inlet three-way valve (Figure 1). The columns were then incubated at room temperature (~23°C) for 2 weeks to establish SRB activity without injection of medium (Callbeck et al., 2011). Subsequently, CSBA-S medium containing 2 mM sulfate and 3 mM VFA was continuously injected into columns at a low flow rate, which was gradually increased to the values indicated in Table 3. The bioreactors were then eluted at this constant flow rate until 1.8–2 mM sulfide were produced in the effluent. The bioreactors were then ready for treatment with biocides and/or nitrate.

**Table 3:** **Dimensions, characteristics and operating conditions of the bioreactors used**.

**Columns**	**BV#**	Column size L^*^D (cm)^a^	**Pore volume (PV, mL)**	**Total volume V (mL)^b^**	**Porosity (%)**	**Flow rate (mL/h)**	**Retention time (h)^c^**	**Velocity (cm/h)^d^**
Plastic	0, 1, 2, 3	12.2^*^2.7	25.7	69.8	36.8	3.7	6.9	1.8
Glass	4, 5, 6, 7, 8	9.8^*^2	10.9	30.8	35.4	2.7	4.0	2.45

a *L is the length and D is the diameter of the bioreactor columns*.

b V = π (D/2)^2^

* *L*.

c *Retention time is pore volume divided by the flow rate*.

d *velocity is L divided by retention time*.

### Biocide and nitrate injection

The biocides used were Glut, BAC and cocodiamine, as well as THPS and Glut_BAC (a mixture containing 42.5% w/w Glut and 7.5% w/w BAC). Glut and BAC were purchased from Sigma-Aldrich and ICN, respectively, whereas Glut_BAC, THPS and cocodiamine were provided by collaborator companies, indicated in the Acknowledgments. All biocides were liquid concentrates except BAC, which was a white solid of which a 50 mM (18,000 ppm) stock solution was made. Biocides were added into the medium directly from concentrates. Biocide concentrations are indicated as ppm of the active ingredient. The bioreactor columns were continuously injected with CSBA-S (2 mM sulfate) or CSBA-SN (2 mM sulfate and 2 mM nitrate) medium. Biocide treatment was initiated by injecting medium with biocide and was stopped by switching back to medium without biocide. The sulfide recovery time (RT), the time needed for recovery of the sulfide concentration to 0.8 to 1 mM (from 1.7 to 2 mM initial sulfide), was determined to represent the inhibition/kill efficacy of biocides (Gardner and Stewart, 2002). The next pulse of biocide treatment was initiated after the sulfide concentration had recovered to 1.7–2 mM for at least 4 days to ensure the complete recovery of SRB activity. Medium with a defined concentration of biocide was injected into 60 ml plastic bioreactor columns for 5 days or in 30 ml glass bioreactor columns for 1 h.

### Chemical analysis

Samples of 0.5 mL were taken from the effluent three-way valve. The concentration of aqueous sulfide was determined immediately using the methylene blue method (Trüper and Schlegel, 1964). High-performance liquid chromatography (HPLC, Waters 600) with an IC-PAK anion column (4.6 × 150 mm, Waters) eluted with 24% v/v acetonitrile, 2% v/v butanol, and 2% v/v borate/gluconate concentrate at a flow rate of 2.0 mL/min was used to detect sulfate with a Waters 423 conductivity detector and nitrite and nitrate with a UV/VIS-2489, Waters detector at 220 nm (Mand et al., 2014).

### Analysis of duplicates

Bioreactors were repeatedly injected with the same biocide (e.g., BV1 and BV4 with Glut only). Injections of a given concentration was usually done only once, i.e., multiple injections were mostly at different concentration. Repeated injections of the same concentration were done as indicated in Table S1. The RTs derived from these differed on average by about 33%. Hence, we will regard differences of two-fold or more as significant, when these are observed at multiple biocide concentrations.

## Results

### Souring control by pulses of biocide in the absence of nitrate

**Figure 2:**
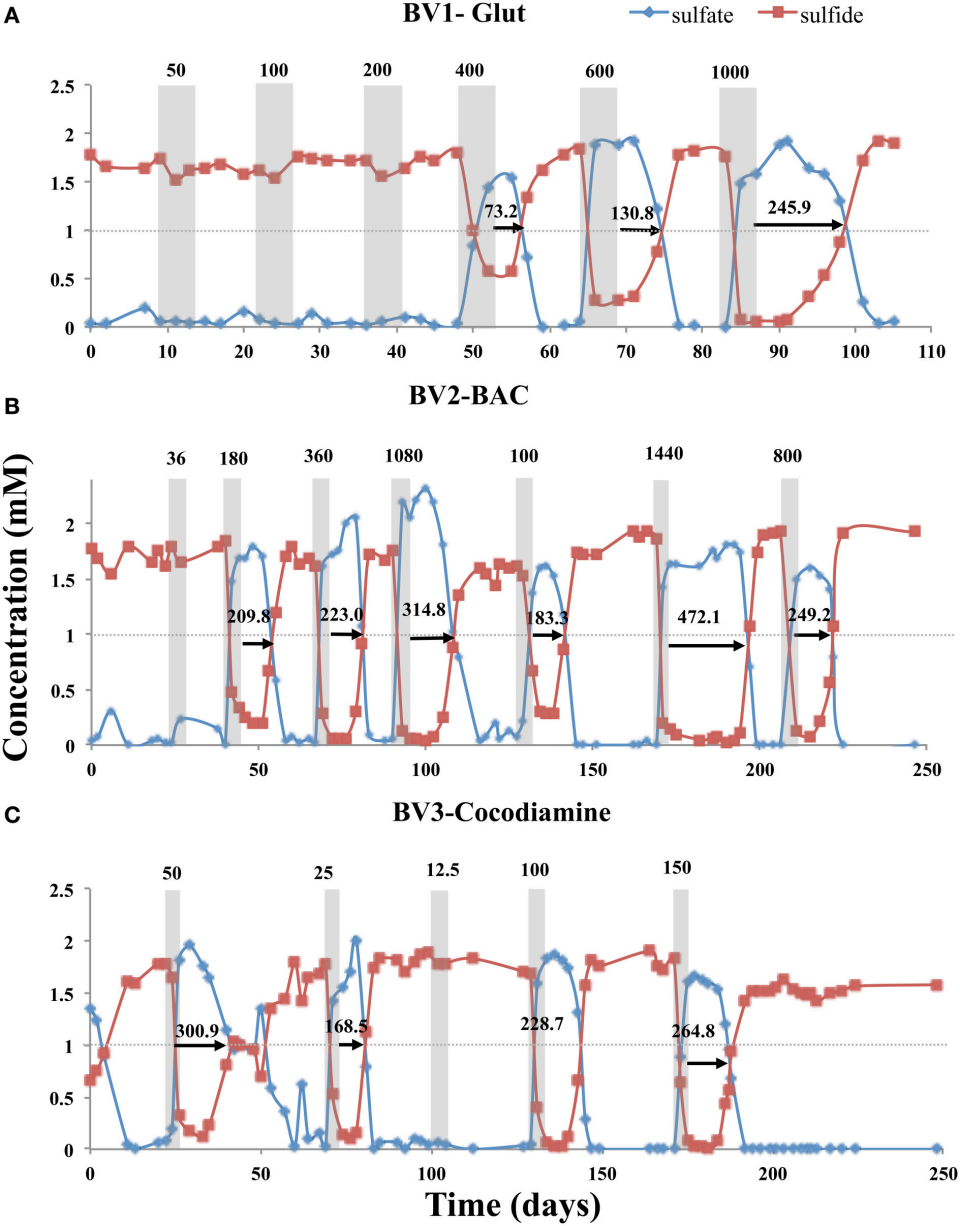
**Effect of 5-day biocide treatment on sulfide production in the absence of nitrate**. Bioreactors BV1, BV2, and BV3 were treated with **(A)** Glut, **(B)** BAC, and **(C)** cocodiamine, respectively. The concentrations of sulfate and sulfide are shown, as indicated. Shaded rectangles indicate the 5-day periods for pulsing biocides. The biocide concentrations (ppm) are indicated above the shaded rectangles. The sulfide recovery times are indicated by the arrows and numbers (h).

Five-day pulses of the biocides Glut, BAC, and cocodiamine were injected into bioreactors BV1, BV2, and BV3, respectively. Injection of 50, 100, or 200 ppm of Glut had no effect on sulfide production in BV1, meaning that RT was 0 h. However, injection of 400, 600, or 1000 ppm gave inhibition of sulfide production with RTs of 73.2, 130.8, and 245.9 h, respectively (Figure 2A, Table S2). Although continuously increasing doses of biocide were applied in the case of Glut, this was not done routinely to avoid adapting the bioreactor community to ever increasing doses of biocide. Bioreactor BV2 was injected with 5-day pulses of 36, 180, 360, 1080, 100, 1440, and 800 ppm of BAC giving RTs of 0, 209.8, 223, 314.8, 183.3, 472.1, and 249.2 h, respectively (Figure 2B). Likewise for bioreactor BV3, application of 50, 25, 12.5, 100, and 150 ppm of cocodiamine gave RTs of 300.9, 168.5, 0, 228.7, and 264.8 h (Figure 2C). A survey of all sulfide RTs obtained for injection of 5-day biocide pulses is given in Figure S1A and Table S2. In the absence of biocide injection, no significant fluctuations in the eluted sulfide concentrations were observed. Bioreactor BV0 injected with CSBA-S medium containing 2 mM sulfate and 3 mM VFA, without biocide, continuously produced 1.78 ± 0.11 mM sulfide (*N* = 70) over 350 days (results not shown).

Bioreactors BV4, BV5, and BV8 were injected with 1 h pulses of Glut, BAC and cocodiamine, respectively, after which the sulfide RT was recorded as indicated in Figure S2A. Injection of 4000, 2000, 1000, 500, 300, and 5000 ppm of Glut gave RTs of 214.6, 135.4, 84.7, 0, 0 and 234.9 h, respectively (Figure S2A, Table S3). This indicated a similar threshold for action of Glut under short-high injection conditions, as compared to long-low injection conditions of 400-500 ppm. The similar threshold may be caused by rapid chemical reaction of Glut with the ammonium in the medium (4.7 mM), inactivating the biocide. Assuming reaction of 1 ammonium/Glut, the calculated threshold is 4.7 mM (470 ppm).

For BAC injection of 500, 1000, 2000, 3000, 3500, and 2500 ppm gave RTs of 0, 0, 0, 250.4, 381.2, and 159.5 h (Figure S2B, Table S3), whereas for cocodiamine injection of 100, 200, 400, 800, 1000, 2000, 3000, and 4000 ppm gave RTs of 0, 0, 0, 0, 0, 40.4, 58.4, and 109.1 h, respectively (Figure S2C, Table S3). Hence, these two biocides appeared to have thresholds in the range of 2000–2500 and 1000–2000 ppm, respectively (Figure S1B, Table S3), whereas under long-low injection conditions these were in the range of 36–100 and 12.5–25 ppm, respectively (Table S2). The effectiveness of the three tested biocides under short-high injection conditions depended on the concentration range used. For concentrations up to 2000 ppm the effectiveness of Glut exceeded that of cocodiamine and BAC, whereas for concentrations of 3000 ppm or higher BAC appeared most effective (Figure S1B).

**Figure 3:**
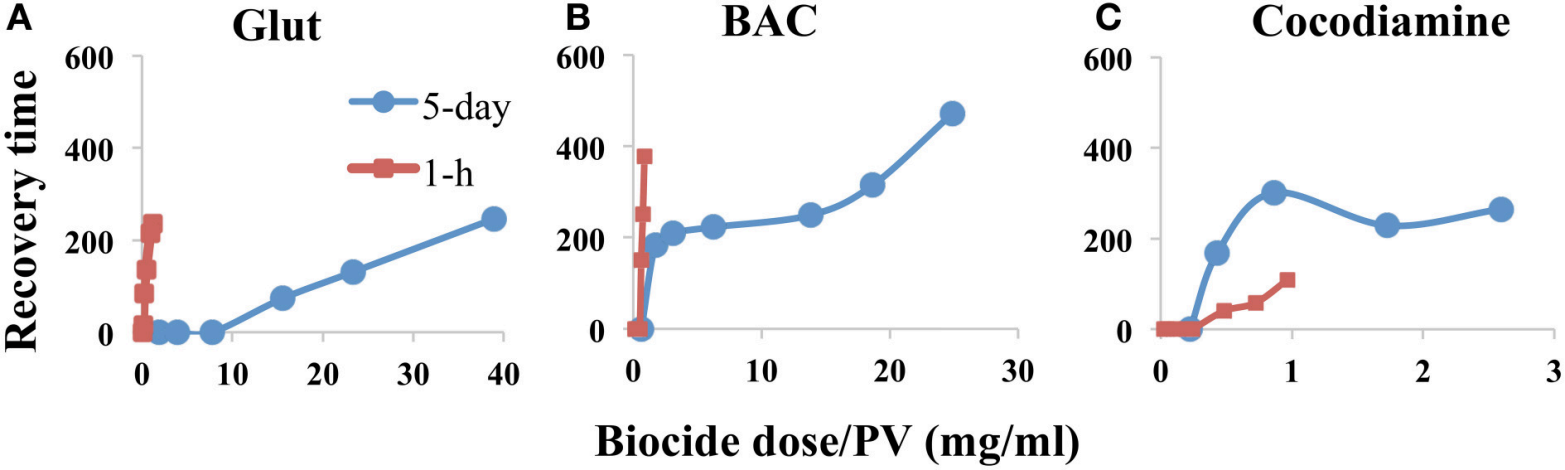
**Sulfide recovery time (h) as a function of biocide dose divided by bioreactor pore volume (mg/ml)**. Data are compared for 5-day and for 1-h pulses of biocide, as indicated, for bioreactors without continuous nitrate injection. Bioreactors were treated with **(A)** Glut, **(B)** BAC, and **(C)** cocodiamine.

In order to more appropriately compare the effectiveness of long-low vs. short-high applications of biocides to inhibit sulfide production from bioreactors, the measured RTs should be plotted against the total amount (mg) of biocide dosed. Because bioreactors with two different PVs were used (Table 3) we divided the total injected amount by the PV, as indicated in Figure 3. The results indicated that the short-high strategy worked best for the fast-acting Glut (Figure 3A). The long-low strategy worked best for the more slowly acting cocodiamine (Figure 3C).

### Souring control by pulses of biocide in the presence of continuous nitrate

**Figure 4:**
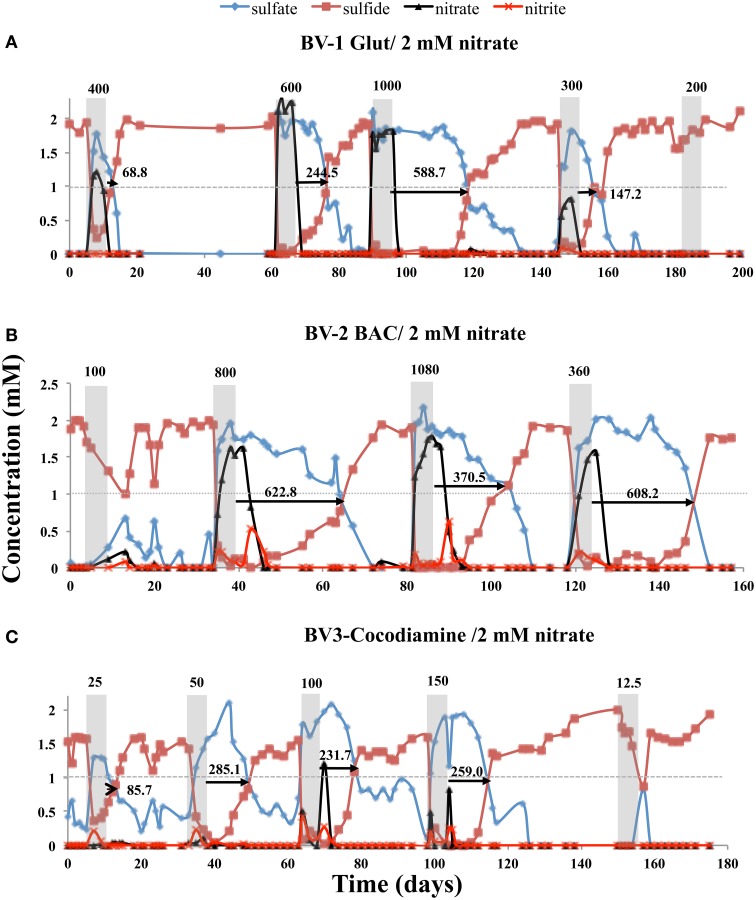
**Effect of 5-day biocide treatment on sulfide production in the presence of nitrate**. Bioreactors BV1, BV2, and BV3 were treated with **(A)** Glut, **(B)** BAC, and **(C)** cocodiamine, respectively. The concentrations of sulfate, sulfide, nitrate and nitrite are shown, as indicated. Shaded rectangles indicate the 5-day periods for pulsing biocides. The biocide concentrations (ppm) are indicated above the shaded rectangles. The sulfide recovery times are indicated by the arrows and numbers (h).

Continuous injection of bioreactors BV1, BV2, and BV3 with CSBA-SN medium, containing 2 mM nitrate and 2 mM sulfate gave complete reduction of both electron acceptors. When the nitrate concentration was increased to 4, 8, or 13.3 mM partial souring control was observed with 8 and 13.3 mM nitrate (results not shown). Following return to injection of CSBA-SN with 2 mM nitrate, 5 day pulses of Glut, BAC or cocodiamine were injected in bioreactors BV1, BV2, and BV3. In addition to concentrations of sulfate and sulfide, those of nitrate and nitrite were also measured. Injection of 400, 600, 1000, 300, and 200 ppm of Glut gave RTs for production of sulfide of 68.8, 244.5, 588.7, 147.2, and 0 h, respectively. This was associated with breakthrough of maximum nitrate concentrations of 1.21, 2.0, 1.82, 0.81, and 0 mM. Only trace nitrite was observed (Figure 4A, Table S2).

When injecting 5-day pulses of 100, 800, 1080, and 360 ppm BAC, RTs of 0, 622.8, 370.5, and 608.2 h were found with peak nitrate and nitrite concentrations of 0.22 and 0.08, 1.63, and 0.53, 1.79, and 0.64, and 1.58 and 0.21 mM, respectively (Figure 4B, Table S2). Injection of 25, 50, 100, 150, and 12.5 ppm of cocodiamine gave RTs of 85.7, 285.1, 231.7, 259.0, and 0 h with peak nitrate and nitrite concentrations of 0 and 0.21, 0.1, and 0.2, 1.2, and 0.4, 0.8, and 0.3, and 0 and 0 mM, respectively (Figure 4C, Table S2).

**Figure 5:**
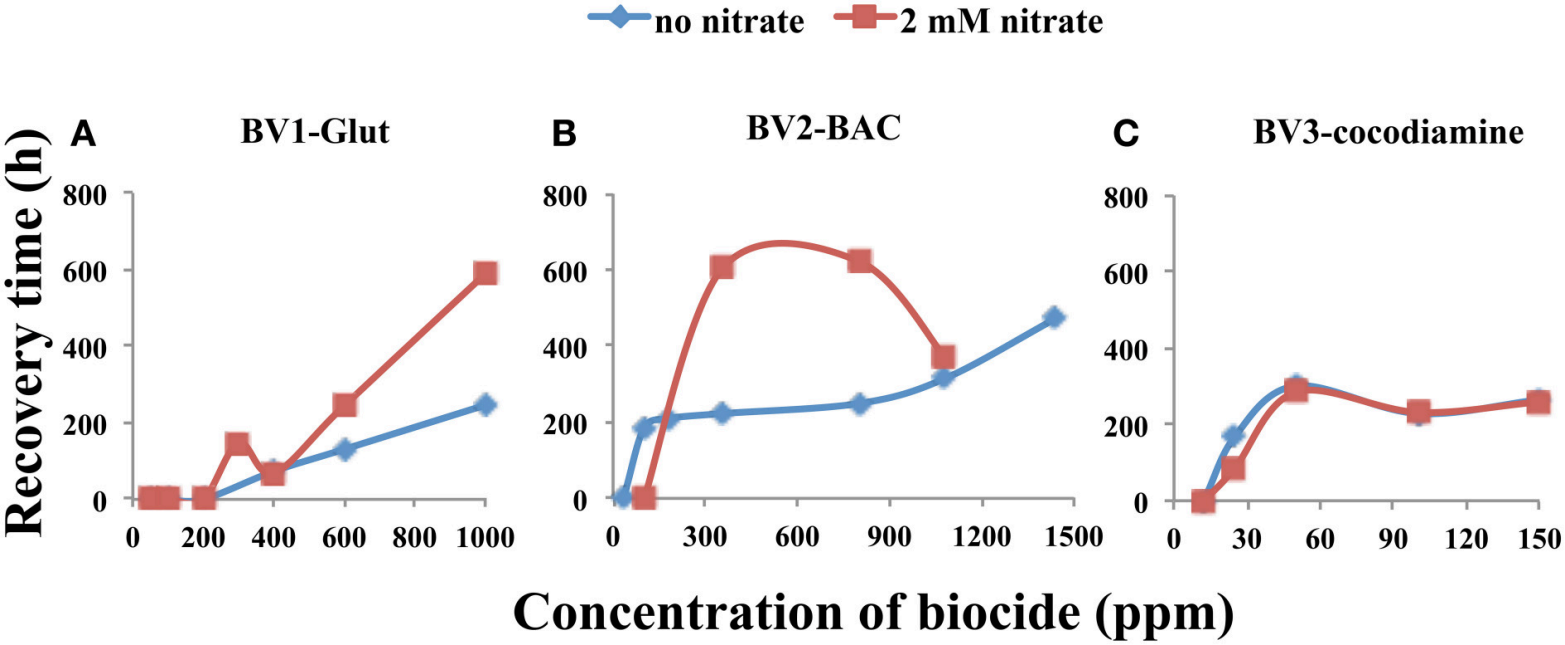
**Relation between sulfide recovery time (RT, h) and biocide concentration for 5-day pulses in the absence and presence of nitrate, as indicated**. Data are for **(A)**, Glut, **(B)**, BAC, and **(C)** cocodiamine.

A comparison of sulfide RTs for 5-day biocide injections in the absence and presence of nitrate is provided in Figure 5. No difference was observed in the case of cocodiamine (Figure 5C). Higher RTs were observed in the presence of nitrate for 360 and 800 ppm BAC (Figure 5B) and for 600 and 1000 ppm of Glut (Figure 5A). In the case of BAC increased production of nitrite could contribute to these increased RTs.

**Figure 6:**
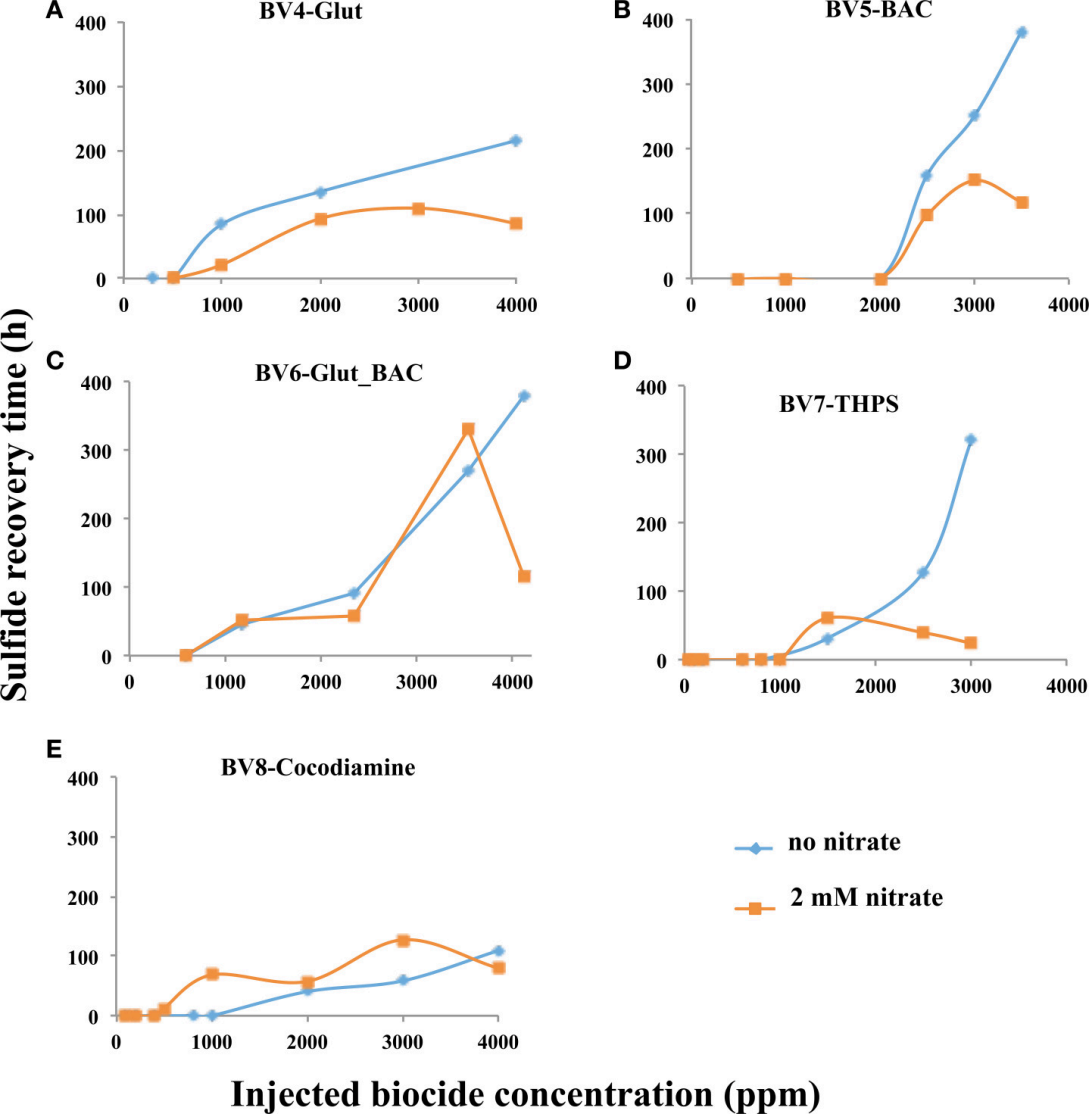
**Relation between sulfide recovery time (RT, h) and biocide concentration for 1-h pulses in the absence and presence of nitrate, as indicated**. Data are for **(A)**, Glut, **(B)**, BAC, **(C)** Glut_BAC, **(D)** THPS, and **(E)** cocodiamine.

Results for pulsing Glut, BAC and cocodiamine for 1 h under continuous injection of 2 mM nitrate are shown in Figures S2, S3. The derived RT values as well as peak nitrate and nitrite concentrations are summarized in Table S3. Injection of 2000, 3000, or 4000 ppm Glut gave breakthrough of 0.21, 0.25, and 0.88 mM of nitrite, respectively (Table S3). Hence, nitrite was observed with Glut (0–0.9 mM), BAC (0–0.43 mM) and cocodiamine (0–0.64 mM). A comparison of derived RT values as a function of biocide concentration in the absence or presence of nitrate is shown in Figure 6. RT values in the presence of nitrate were smaller than in its absence for injections of Glut and BAC, but not for injections of cocodiamine (Figures 6A,B,E).

### Use of other biocides: Glut_BAC and THPS

The effects of injection of Glut_BAC or THPS were only studied for 1-h pulses in the absence or presence of nitrate. The results are summarized in Table S3. Injection of up to 4000 ppm of Glut_BAC and of up to 3000 ppm of THPS increased RT to similar values of 300–400 h, as observed for Glut and BAC (Figure 6). For bioreactors with nitrate up to 0.87 mM of nitrite was produced during injections with Glut_BAC, but less (up to 0.24 mM) during injections with THPS. RT values for injections with these biocides were similar or lower for bioreactors with nitrate, than for bioreactors without nitrate (Figures 6C,D).

## Discussion

The concentrations of biocides needed to control SRB activity in bioreactors in this study were high with hundreds of ppm needed for 5-day and thousands of ppm needed for 1-h pulses (Figures 5, 6), which is higher than for previous studies (Reinsel et al., 1996; Baudrion et al., 2000; Bartlett and Kramer, 2011; Moore and Cripps, 2012). The likely explanation is that due to the continuous injection of medium highly active SRB populations grew in the bioreactor columns as biofilms, which have higher resistance to biocides than planktonic cells (Baudrion et al., 2000; Gardner and Stewart, 2002). The tightly packed sand grains within the bioreactor columns provided a much larger surface area than coupon surfaces in a Robbins device, which is often used to study biocide resistance of biofilms (Grobe and Stewart, 2000). This may also explain the high biocide concentrations needed. When comparing the doses used for the two treatment strategies in the absence of nitrate, it appeared that the fast-acting Glut performed better during 1-h injections at high concentration, whereas the more slowly acting cocodiamine performed best during 5-day injections at lower concentration (Figure 3).

When nitrate is reduced by NRB it inhibits SRB by competitive exclusion. NRB grow faster, to a higher cell density and at a higher redox potential than SRB. Moreover they produce nitrite, which is a strong SRB inhibitor (Reinsel et al., 1996; Sturman et al., 1999; Haveman et al., 2004). Hence if electron donor (e.g., VFA) is limiting, we expect partial reduction of nitrate with accumulation of nitrite and permanent inhibition of SRB. However, if electron donor is in excess, as in the present study, the outcome will be different. When a consortium of NRB and SRB was inoculated into a serum bottle, containing medium with nitrate, sulfate and heavy MHGC oil as excess electron donor, then nitrate was reduced first, followed by reduction of sulfate, which in turn was followed by methanogenesis (Agrawal et al., 2012). In bioreactors injected with limiting nitrate and sulfate and excess VFA, or containing excess heavy oil, these temporal zones are also spatially separated causing a zone of nitrate reduction near the bioreactor inlet to be followed by a zone of sulfate reduction further downstream (Callbeck et al., 2011, 2013). The NRB biomass near the inlet may exceed the SRB biomass further downstream by an order of magnitude, because nitrate reduction yields much more energy than sulfate reduction (Table 2).

The use of multiple agents to control sulfidogenesis has indicated synergy between two types of biocides (Al-Hashem et al., 1998; Greene et al., 2006; McGinley and Van Der Kraan, 2013), a biocide and a metabolic inhibitor (nitrite or molybdate) (Greene et al., 2006), and two types of metabolic inhibitors (Mustafa and Shahinoor Islam Dulal, 1996; Nemati et al., 2001b; AI-Refaie et al., 2009). However, the combined effect of biocide and nitrate on souring control has not been studied, likely because nitrate only becomes inhibitory to SRB following its reduction to nitrite. The inhibitory effect of nitrite on SRB has been extensively studied (Reinsel et al., 1996; Sturman et al., 1999; Greene et al., 2003; Haveman et al., 2004). As an analog of sulfite, nitrite binds to dissimilatory sulfite reductase (Dsr), preventing sulfide production. Additionally, nitrite can chemically react with sulfide forming N_2_ and elemental sulfur (Reinsel et al., 1996).

When a biocide is pulse-injected into a zoned bioreactor the NRB biomass may protect the SRB biomass from killing by the biocide, depending on the mechanism of action of the biocide (Table 1). Glut is a cross-linking agent that irreversibly reacts with amino groups of proteins and nucleic acids, while BAC and cocodiamine are quaternary cationic surfactants that form micelles and can physically interact with cell membranes, causing rupture of the cells (Greene et al., 2006). Because NRB are located closer to the biocide injection point, NRB biomass may protect SRB biomass from chemical attack by Glut. However, in the case of physically interacting biocides it is less clear whether such protection is possible. If, in a zoned system, BAC or cocodiamine bind to and kill NRB biomass these could subsequently interact with and kill SRB biomass. On the other hand SRB biomass may become more sensitive to biocide, if its action on NRB leads to accumulation of nitrite. Nitrite accumulation may occur if the activity of nitrate reductase, reducing nitrate to nitrite, is less affected by the biocide than the activity of enzymes acting in the reduction of nitrite to N_2_. Such differential action could be caused, for instance, by the fact that nitrate reductase is often cytoplasmic-membrane bound, whereas nitrite-, NO- and N_2_O-reductase are periplasmic-membrane bound (Zumft, 1997). The latter may thus be more easily accessed by biocides. Indeed breakthrough of nitrite (up to 0.8 mM, 40% of injected nitrate) during biocide treatment was observed with all biocides (Tables S2, S3) with lower values being observed for THPS (Table S3: up to 0.24 mM nitrite). Greene et al. (2006) investigated the effect of combined addition of nitrite and biocide and found that sulfide production by SRB was synergistically inhibited by nitrite and Glut, BAC, cocodiamine or bronopol, but not by nitrite and THPS, which was thought to chemically react with nitrite.

Hence, synergy between continuously injected nitrate and pulsed biocide is possible but it is hard to predict. It was observed for short-high concentration pulses of cocodiamine (Figure 6E) and for long-low concentration pulses of Glut and BAC (Figures 5A,B). Synergy is expected for a compound, which strongly inhibits the reduction of nitrite without affecting the reduction of nitrate. All biocides tested had this property to some extent. Further work should, therefore, concentrate on finding agents, which are better at this than those tested so far.

## Author contributions

YX contributed to setting up the experiments as designed, collecting all data and interpretation of the data and also contributed to the writing of the manuscript during all stages. GV advised on experimental design and operation, interpretation of the data and writing of the final manuscript.

### Conflict of interest statement

The authors declare that the research was conducted in the absence of any commercial or financial relationships that could be construed as a potential conflict of interest.
