# Supplementary material for: Control of Microbial Sulfide Production with Biocides and Nitrate in Oil Reservoir Simulating Bioreactors
Source: Front Microbiol. 2015 Dec 8;6:1387. doi: 10.3389/fmicb.2015.01387 (PMC4672050; doi:10.3389/fmicb.2015.01387)
Supplement: Supplementary file 1 [file DataSheet1.DOCX]

***Supplementary Material***

**Control of Microbial Sulfide Production with Biocides and Nitrate in Oil Reservoir Simulating Bioreactors**

**Yuan Xue, Gerrit Voordouw***

Petroleum Microbiology Research Group, Department of Biological Sciences, University of Calgary, Calgary, AB, Canada

*** Correspondence:** Gerrit Voordouw, Petroleum Microbiology Research Group, Department of Biological Sciences, University of Calgary, 2500 University Drive NW, Calgary, AB, T2N 1N4, Canada.

[voordouw@ucalgary.ca](mailto:voordouw@ucalgary.ca)

**Table S1.** Recovery times (RT) for bioreactors that were treated twice with the same biocide concentration. The average RT, the range of the two values (in h and in % ) are indicated.

| Bioreactor | Nitrate (mM) | Biocide | C (ppm) | RT1 (h) | RT2 (h) | Average (h) | Range (h) | Range (%) |
| --- | --- | --- | --- | --- | --- | --- | --- | --- |
| BV3 | 2 | Cocodiamine | 25 | 89.2 | 85.7 | 87.45 | 3.5 | 4.00 |
| BV5 | 2 | BAC | 3500 | 117.7 | 123.2 | 120.45 | 5.5 | 4.57 |
| BV6 | 0 | Glut_BAC | 3531 | 92.3 | 269.7 | 181 | 177.4 | 98.01 |
| BV7 | 0 | THPS | 2500 | 94 | 128.2 | 111.1 | 34.2 | 30.78 |
| BV8 | 2 | Cocodiamine | 2000 | 76.6 | 57.5 | 67.05 | 19.1 | 28.49 |
|  |  |  |  |  |  |  | Average | 33.17 |

**Table S2.** Summary of determined values of sulfide recovery times RT for bioreactors treated with 5-day pulses of biocide. For data obtained in the presence of nitrate the peak nitrate and nitrite concentrations are also given.

*added from 50 mM BAC stock solution

†C= biocide concentration;

“RT=0” means that no inhibition of sulfide production was observed;

“N/A” indicates not applicable because this condition was not tested.

**Table S3.** Summary of determined values of sulfide recovery times RT for bioreactors treated with 1-h pulses of biocide. For data obtained in the presence of nitrate the peak nitrate and nitrite concentrations are also given.

*C= biocide concentration;

“RT=0” means that no inhibition of sulfide production was observed;

“N/A” means not applicable, because this condition was not tested.

**Figure S1⏐Relation between sulfide recovery time (RT) and biocide concentration (ppm) for (A) 5-day and (B) 1-h pulsed treatments.** Both are in the absence of nitrate.

**Figure S2⏐Effect of 1-h biocide treatment on sulfide production in the absence of nitrate.** Bioreactors BV4, BV5 and BV8 were treated with (A) Glut, (B) BAC and (C) cocodiamine, respectively. Dotted lines indicate the 1-h periods for pulsing the indicated concentrations of biocide (ppm). The sulfide recovery times are indicated by the arrows and numbers (h).

**Figure S3⏐Effect of 1-h biocide treatment on sulfide production in the presence of nitrate.** Bioreactors BV4, BV5 and BV8 were treated with (A) Glut, (B) BAC and (C) cocodiamine, respectively. Dotted lines indicate the 1-h periods for pulsing the indicated concentrations of biocide (ppm). The sulfide recovery times are indicated by the arrows and numbers (h).
